# Tracing the Cognitive–Motor Connection: Prospective-Longitudinal Associations Between Early Parent–Toddler Literacy Activities and Subsequent Gross Motor Skills at School Entry

**DOI:** 10.3390/children12111431

**Published:** 2025-10-23

**Authors:** Nairy Kazandjian, Kianoush Harandian, Marie-Michèle Dufour, Elena A. Chichinina, Michel Desmurget, Linda S. Pagani

**Affiliations:** 1 School of Psycho-Education, Université de Montréal, C.P. 6128, Montreal, QC H3C 3J7, Canada; nairy.kazandjian@umontreal.ca (N.K.); kianoush.harandian@umontreal.ca (K.H.); marie-michele.dufour@umontreal.ca (M.-M.D.); 2 School Environment Research Group, Université de Montréal, Montreal, QC H3T 1J4, Canada; 3 Sainte-Justine’s Pediatric Hospital Research Center, Université de Montréal, Montreal, QC H3T 1J4, Canada; 4 Faculty of Psychology, Lomonosov Moscow State University, Moscow 119991, Russia; 5 Institut des Sciences Cognitives Marc Jeannerod, Université de Lyon, 69361 Bron, France; michel.desmurget@inserm.fr

**Keywords:** literacy stimulation, motor development, motor skills, sensitive periods, embodied cognition, parent–child activities, child development

## Abstract

**Highlights:**

This study examines the long-term relationship between early literacy stimulation and later motor development, highlighting how early parent–child reading and writing experiences influence motor outcomes.

**What are the main findings?**

- This study found longitudinal associations between toddler literacy stimulation and higher gross motor skills scores by age 6 among girls.
- No significant associations were found for boys.

**What are the implications of the main findings?**

- Biological and environmental factors may account for the variation in motor outcomes between girls and boys.
- Early childhood policies should expand equitable access to books, and guide parents to enhance literacy routines (reading stories, tracing words, acting out narratives) to support both cognitive and motor development.

**Abstract:**

**Background/objectives**: Early literacy is widely promoted, yet its broader developmental benefits remain underexamined regarding key indicators of brain development. This study examines whether early literacy exposure in toddlerhood predicts motor skill development at the end of kindergarten. **Methods**: Participants comprised 1006 boys and 991 girls from the Quebec Longitudinal Study of Child Development (QLSCD) birth cohort. Early literacy stimulation was measured at age 2 years using parent reports of frequency of shared reading, looking at books or comics, and pre-writing activities such as scribbling and tracing. At age 6 years, child motor development was assessed by trained examiners. Sex-stratified multiple regression models were examined, adjusting for pre-existing and concurrent child and family characteristics. **Results**: Early literacy stimulation was significantly associated with better motor control skills among girls (β = 0.10, *p* < 0.05). For boys, a non-significant positive trend was observed for both motor and locomotion skills. **Conclusions**: Our findings underscore the lasting influence of early literacy stimulation and subsequent motor skills—particularly for girls who may receive less gross motor encouragement than boys. As such, promoting literacy-rich environments in toddlerhood is a family strategy to support healthy, confident, and active youth development.

## 1. Introduction

Early literacy, which includes activities such as shared reading, storytelling and scribbling, is widely promoted and recognized as a foundational element of school readiness and healthy child development [1]. Surprisingly, it remains underexamined through rigorous longitudinal research. While many have examined long-term cognitive skills, important gaps remain in our understanding of the broader developmental impacts of early literacy stimulation across diverse contexts [2,3,4]. Among these underexplored domains are fine and gross motor skills, which are essential for children’s physical activity, development, and everyday functioning [5].

Motor skills encompass both fine and gross domains. Fine motor skills include reaching, grasping, and manipulating objects, whereas gross motor skills involve static positioning, quality of movement (e.g., running, jumping, catching), and motor planning [6]. Studies have shown that early motor skills are powerful predictors of later academic achievement [7]. Research on school readiness finds that both fine and gross motor proficiency at kindergarten forecasts subsequent reading and math comprehension in third [8] and fourth grade [9]. A recent randomized intervention in Hong Kong revealed that improving object control and fundamental movement skills in 3 to 5 year olds led to significant gains in executive function, which are key for later academic success [10]. Moreover, a longitudinal analysis of 3188 children from the UK Millennium Cohort study aged 9 months to 11 years concluded that gross motor skills at 9 months were positively associated with spatial working memory, while early fine motor skills predicted later performance in English and science [11].

Early literacy enrichment, such as shared book reading, storytelling, and exposure to print, predicts a wide range of long-term outcomes, including language development, school readiness, and academic engagement and achievement [12,13]. Prospective-longitudinal studies in children from different cultures have consistently shown that early literacy stimulation enhances later vocabulary, reading comprehension, and even socioemotional adjustment [14,15]. Furthermore, early literacy experiences typically unfold in emotionally supportive interactions, such as shared reading or play, which promote self-regulation and motivation, which are key precursors of coordinated cognitive and motor development [16]. These interactions may therefore contribute indirectly to the development of integrated brain systems that support learning and coordination across domains [16]. These findings underscore how early environments shape developmental trajectories well into adolescence. Curiously, because motor development is often sidelined in human development research, very little is known about how early literacy exposure might influence later motor skills.

From a theoretical lens, the first 1000 days suggests that early life represents a biologically sensitive window during which experiences can have disproportionate and lasting effects on development [17]. This sensitive period is described by Gee & Cohodes [18] as a period of heightened neuroplasticity during which environmental inputs can exert an especially powerful influence on long-term developmental outcomes. Family experiences during this period, such as shared literacy-related activities, may help foster the learning-related behavioral mechanisms that underlie knowledge acquisition. Indeed, exposure to literacy during this period may shape neural architecture in ways that extend beyond language development alone.

Neuroimaging studies show that early literacy experiences engage and strengthen networks that integrate auditory, visual, and motor systems, particularly within the left temporoparietal cortex, inferior frontal gyrus, and occipitotemporal regions associated with visual–phonological mapping and print–speech integration [19]. Early shared reading and print exposure have also been linked to increased white-matter organization and connectivity between frontal and temporal regions supporting executive function and self-regulation [20,21]. These findings suggest that literacy-related experiences in the early years contribute to the maturation of multimodal brain networks that support not only language but also cognitive control, attention, and motor planning.

Complementing this is Piaget’s classic theory of cognitive development, which posits that learning is an active process of constructing knowledge through sensorimotor interaction with the environment [22]. Early development unfolds through sensorimotor exploration, where bodily actions and perceptual experiences serve as the foundation of cognition [23,24]. From this perspective, early literacy activities such as turning pages, pointing at pictures, gesturing, and acting out stories, or scribbling are not just cognitive exercises but embodied sensorimotor experiences that stimulate multiple developmental domains, including motor functioning. They require fine motor coordination, hand–eye integration, and visuospatial planning, which directly contribute to motor skill refinement during early childhood [8]. Thus, literacy experiences are not merely linguistic or symbolic acts but embodied learning opportunities that integrate action, perception, and cognition within meaningful social contexts. Much of these experiences originate in early childhood [12,13].

Building on this foundation, embodied cognition frameworks extend Piaget’s ideas by suggesting that higher-order thinking is grounded in sensorimotor systems [24]. These frameworks propose that language, literacy, and motor behavior rely on partially overlapping neural circuits, particularly within the cerebellum, premotor cortex, and frontoparietal networks that support both movement and symbolic processing [25,26]. Recent perspectives that leverage embodied cognition frameworks suggest a bidirectional relationship between motor development and executive functioning [11,27]. Motor skills serve as a developmental scaffold for cognitive growth and vice versa. In this view, motor skills not only support the development of cognitive processes, acting as a scaffold for emerging executive functions, but maturing executive functions can also enhance motor control and coordination, especially in complex or goal-directed tasks. This reciprocal dynamic reflects the brain’s integrative architecture, where regions responsible for motor planning and coordination (e.g., cerebellum, basal ganglia) also contribute to cognitive functions such as working memory, inhibition, and attentional control [27].

Importantly, early literacy activities such as shared reading, counting, and guided play have been associated with higher executive functioning [28]. This supports the idea that participation in such cognitively stimulating activities may indirectly contribute to improved motor skills through the reciprocal pathway between executive functioning and motor development. This link is further supported by neurodevelopmental findings that show parallel maturation and co-activation of the cerebellum (involved in motor control), and prefrontal cortex associated with cognitive tasks [29]. Taken together, the evidence suggests that early interactions during learning moments, such as during literacy-related activities, play a key role in shaping emerging cognitive capacities and, in turn, can help foster the development of motor skills. These overlapping neural pathways support the idea that motor and cognitive systems develop in tandem, influencing one another across early childhood [27]. Consequently, early literacy experiences that combine verbal, visual, and motor engagement—such as shared reading, storytelling, and scribbling—may strengthen executive functioning and motor control simultaneously. Through this embodied mechanism, literacy stimulation may shape the neural and behavioral architecture that underlies both academic and motor development [30,31].

Previous research in this area presents several methodological limitations. First, the predominance of cross-sectional designs offers only a snapshot of development, limiting our ability to trace behavioral trajectories over time [8]. Second, many studies fail to account for critical confounding factors beyond maternal education or income, such as early cognitive functioning, temperament, or parental mental health, which may independently shape both early experiences and later outcomes [32,33]. Third, most studies treat sex as a covariate rather than examining sex-specific patterns, thereby overlooking the distinct biological and social contexts in which boys and girls develop. Stratified analyses allow for a more accurate depiction of development by acknowledging that boys and girls may respond differently to environmental inputs due to both inherent and socially constructed differences [34,35]. Gender socialization theory suggests that individuals learn the behaviors, roles, attitudes, and expectations that society associates with being male or female [36]. Parental roles play a central part in this process, especially in early childhood, when parents model gendered behaviors and influence children’s preferences, activities, and social interactions [6]. Through the toys they provide, the expectations they communicate, and the division of labor they model at home, parents help shape children’s understanding of what is considered appropriate for each gender [6,36].

Using a population-based, millennial birth cohort of boys and girls, this study aims to investigate prospective associations between literacy stimulation in toddlerhood and motor skill development at school entry. Specifically, we examined whether parent-reported early exposure to books, writing related activities, and shared reading at age 2 years predicts motor functioning at age 6 years in typically developing children. Drawing on theories of sensitive periods, embodied cognition, and developmental constructivism, we hypothesized that higher levels of early literacy stimulation would predict higher motor skill development by promoting repeated, structured, and meaningful motor actions within a socially embedded learning context. 

## 2. Methods

### 2.1. Participants

Participants from this IRB-approved investigation are from the Quebec Longitudinal Study of Child Development (QLSCD) birth cohort, coordinated by the *Institut de la Statistique du Québec*. The original sample included 2837 infants born between spring 1997 and 1998 across all administrative regions of Quebec, Canada, and was designed to track development outcomes in a representative group of typically developing children. Of these, 93 selected children were excluded due to ineligibility, 172 could not be located, 14 were unreachable, and 438 declined participation. This resulted in a final cohort of 2120 infants eligible for initial follow-up beginning at age 5 months [37]. Data collection occurred annually in early childhood. Informed consent was obtained from parents at each wave. For the present study, we retained a subsample of 1006 boys and 991 girls with complete data on literacy exposure at age 2 years. 

### 2.2. Measures: Predictor—Literacy Exposure (Age 2 Years)

In early childhood, parents reported on informal home literacy environment, which are unstructured frequency-based opportunities within a family that encourages reading development [38,39,40]. Specifically, they were asked about the frequency of the types of literacy stimulation their child was exposed to in the home: (a) looked at books, magazines, comics, etc., (b) played with pencils or markers doing real or pretend writing at home, and (c) was read to by an adult of the household. Responses varied on an 8-point Likert scale from “Never or rarely” to “Many times each day”, recoded into “Once a week or less” (=0) or “A few times a week or more” (=1) for each question. Scores for each of the three items were summed to produce a composite index of home literacy exposure, with possible values ranging from 0 to 3.

### 2.3. Measures: Outcomes—Gross Motor Skills (Age 6 Years)

Object control skills and locomotion skills were assessed by trained examiners using the Test of Gross Motor Development 2nd edition [41]. The assessment includes two subtests: The Object Control Subtest, composed of six tasks (striking a stationary ball, stationary dribble, catch, kick, overhand throw, underhand roll), and the Locomotor subtest, composed of six tasks (run, gallop, hop, leap, horizontal jump, slide). For each task, the examiner first demonstrated the motor action, then the child was asked to repeat the action twice. One point was given for each performance criterion demonstrated successfully, for a total of 12 points for each subtest. For each skill, a sum of scores was calculated and standardized.

### 2.4. Measures: Confound Control Variables (Ages 5 Months to 2 Years)

Key individual and family characteristics from early childhood were examined to account for potential pre-existing or simultaneous factors that could confound the relationship between early literacy stimulation and later motor control or locomotor skills.

In terms of childhood-related variables, at age 5 months, mothers reported gestational smoking or substance use (0 = no substance use, 1 = any substance use), whereas premature birth (0 = no preterm, 1 = preterm at less than 37 weeks) and weight for gestational age (g/week) were obtained from medical records. The Infant Characteristics Questionnaire was used to report child temperament problems at age 1.5 years (10 items; 0 = below the median, 1 = above the median; α_Boys_ = 0.78 and α_Girls_ = 0.75; [42]). Neurocognitive skills were assessed by trained examiners at age 2 years using the Imitation Sorting Task [43].

Regarding the potential family-related confounds, maternal antisocial antecedents were identified at age 5 months using the National Institute of Mental Health—Diagnostic Interview Schedule (9 items; 0 = below the median, 1 = above the median; α = 0.46; [44]). Mothers also reported on maternal education at age 5 months (0= finished high school, 1 = did not finish high school), and maternal depressive symptoms using the Center for Epidemiologic Studies Depression Scale at age 1.5 years (13 items; 0 = below the median, 1 = above the median, α = 0.82; [37]). Parents reported family dysfunction using the McMaster Family Assessment Device at age 5 months (12 items; 0 = below the median, 1 = above the median; α 0.88; [45]), family income at age 2 years (0 = sufficient income, 1—insufficient income; as defined by the Canadian low-income cut-off of that year provided by Statistic Canada [31], and family configuration at age 2 years (0 = intact; 1 = non-intact). 

### 2.5. Data Analytic Procedures

Statistical analyses were conducted using SPSS (v.29) and Mplus (v.8.0). Longitudinal prospective linear associations were estimated using sex-stratified ordinary least squares multiple regression models. Indicators of motor control skills and locomotion skills at age 6 years (MOTOR_i,age6_) were regressed on literacy stimulation in the home at age 2 years (LIT_i,age2_). To account for potential omitted variable bias and alternative explanations, the models included a comprehensive set of pre-existing and concurrent child (CHILD_iages5mo–age2_) and family characteristics (FAMILY _iages5mo–age2_) as covariates. All predictors and controls were entered simultaneously into one fully adjusted model, where “a” represents the intercept and “e” the stochastic error:

MOTOR_i,age 6_ = a + β1LIT_i,age 2_ + γ1CHILD_i,ages5mo–age2_ + γ2FAMILY_i,ages5mo–age2_ + e_i_

As is typical in longitudinal designs, some participants had incomplete data across different waves. To address this, we used Full Information Maximum Likelihood (FIML) estimation with robust standard errors (MLR) in Mplus, which allows for the inclusion of all available data and corrects for potential attrition bias. 

## 3. Results

Table 1 reports descriptive statistics for the predictor, outcome, and control variables between the ages of 5 months and 6 years. Notably, by age 2 years, more than half of the boys (54.7%) and girls (57.3%) had been exposed to all three forms of literacy stimulation in the home. Fewer than one in ten children were exposed to one or fewer forms of stimulation. At age 6 years, girls slightly outperformed boys in both motor control and locomotion skills, with average scores near the midpoint of the range for both sexes.

Table 2 documents the adjusted unstandardized regression coefficients estimating the relationship between child and family characteristics (measured between 5 months and 2 years) and literacy exposure at age 2 years, separately for boys and girls. Among boys, lower maternal education (β = −0.09, *p* < 0.05, 95% confidence interval [CI]: −0.32, −0.04) and higher levels of family dysfunction (β = −0.06, *p* < 0.05, 95% CI: −0.19, −0.01) were significantly associated with reduced literacy exposure. Among girls, being born prematurely (β = −0.08, *p* < 0.05, 95% CI: −0.46, −0.002), higher maternal depressive symptoms (β = −0.09, *p* < 0.05, 95% CI: −0.19, −0.02), and greater neurocognitive skills (β = 0.12, *p* < 0.001, 95% CI: 0.04, 0.13) significantly predicted literacy exposure. 

Table 3 reports adjusted unstandardized regression coefficients reflecting the relationship between literacy stimulation at age 2 years and motor development by age 6 years for boys and girls. For girls, literacy stimulation at age 2 years was significantly associated with better motor control skills at age 6 years (β = 0.10, *p* < 0.05, 95% CI: 0.10, 0.82). Every unit increase in shared reading and writing exposure predicted a 15.6% unit increase in motor control skills. A non-significant trend was observed for locomotion skills (β = 0.07, *p* = 0.071, 95% CI: −0.03, 0.76). For boys, early literacy exposure was not associated with better motor outcomes and locomotion skills.

Several early individual and family characteristics also predicted motor outcomes. For girls, family dysfunction surprisingly predicted better motor control (β = 0.09, *p* < 0.05, 95% CI: 0.02, 0.96), while lower income was linked to lower motor outcomes (β = −0.13, *p* < 0.05, 95% CI: −1.59, −0.15). For boys, family dysfunction (locomotion: β = −0.09, *p* < 0.05, 95% CI: −1.04, −0.02) and lower family income (motor: β = −0.15, *p* < 0.05, 95% CI: −1.61, −0.21; locomotion: β = −0.13, *p* < 0.05, 95% CI: −1.76, −0.09) were significantly associated with lower motor skills.

## 4. Discussion

Reading to very young children is widely recognized as a foundational practice that supports language development, cognitive growth, and school readiness [46]. Our findings contribute to this growing area of research by providing timely evidence of associations between early literacy stimulation and later motor outcomes in typically developing boys and girls, extending through to the start of kindergarten. Motor skills are not only an indicator of healthy brain development but also forecast academic achievement, particularly in math and reading outcomes [36,47].

Our findings suggest that just over half of toddlers were exposed to all three types of literacy stimulation at home. These experiences consist of being read to, storytelling, and having access to books or printed materials with an adult caregiver at home. Early exposure to these cognitively engaging and hands-on fine motor activities was generally positive, though it varied across families and did not predict outcomes equally for boys and girls.

For females, experiencing literacy stimulation in toddlerhood predicted better motor control skills at kindergarten, compared with females who did not experience early literacy stimulation. More specifically, increases in shared reading and exposure to the printed word between adult and child at age 2 years predicted subsequent increases in motor skills at age 6 years. This suggests they were more likely to perform better on tasks requiring coordination, striking a stationary ball, stationary dribble, catch, kick, overhand throw, underhand roll than their same-sex peers with less early literacy experiences. This relationship remained even after accounting for potential confounders.

By contrast, we observed no meaningful relationship between literacy stimulation in toddlerhood and subsequent motor skills in boys. This means that boys who experienced early literacy enrichment did not perform better on tasks such as catching, kicking, running and side shuffling than their same-sex peers with less literacy stimulation experience as toddlers. We also did not observe a relationship between literacy exposure and locomotion skills in girls. 

Gender Socialization Theory may help explain why girls appeared more responsive to literacy stimulation, showing stronger gains in motor control skills than boys by age 6 years. This theory suggests that parents shape child behaviors and beliefs through modeling and structuring of daily activities [48]. Although both boys and girls in our sample received similar forms of literacy stimulation, such as shared reading and pre-writing activities, the broader socialization context may shape how these experiences are integrated developmentally. Environmental factors often shape the experiences of boys and girls differently, including the nature of parent–child interactions around literacy [49]. For instance, girls are often socialized into quieter, fine-motor-focused activities such as reading and writing. In this context, tasks that combine language and specific motor skills, such as drawing pointing, crafting, or writing may help foster motor development, which could explain the stronger association observed between early literacy stimulation and motor development in girls. 

In agreement with this view, a recent longitudinal study by Bowler et al. [48] provides compelling evidence that early fine motor activities, such as drawing, may scaffold neuromotor pathways that later support gross motor coordination. These findings reinforce the view that motor domains are interrelated, and early fine motor practice can enhance whole-body movement fluency later on. Literacy-rich environments give children valuable opportunities to enhance hand–eye coordination, a fundamental pillar of motor skill development, especially when reading is integrated with activities like drawing, writing, or interactive storytelling [50]. Furthermore, research suggests that pairing literacy with physical movement, such as gestures, tracing letters, or acting out story elements can strengthen both emergent literacy skills and motor development. Everyday literacy practices like turning pages, pointing to pictures, or tracing shapes directly activate fine motor processes, laying a foundation for more complex motor competencies later in childhood [51].

In contrast, although boys in this study received similar levels of literacy stimulation, they have a natural early childhood advantage in object control skills and are typically exposed to more gross motor stimulation early in life and receive more encouragement and verbal cues aimed specifically at promoting gross motor development from a young age [6]. This emphasizes that parental behaviors significantly shape early motor skill development, effectively amplifying innate gender differences through socialization. These differences reflect broader family perceptions of which activities are considered appropriate or beneficial for each gender, shaping both the quantity and type of early stimulation children receive. Therefore, the fact that boys may already demonstrate stronger gross motor skills earlier in development and receive more encouragement can help explain the nonsignificant relationships for boys in response to early literacy stimulation. 

These findings highlight the benefits of early literacy stimulation extending beyond cognitive and academic outcomes to include motor development. They support the view that early learning experiences interact dynamically across developmental domains. When young children engage in shared reading, scribbling, or exploring print, they are not only building vocabulary and comprehension but also working on their fine motor control and coordination. These everyday literacy practices involve purposeful movement embedded within a social context, which may explain why girls who are socialized toward these quieter, detail-oriented tasks demonstrate greater gains in motor control, compared to girls who are not.

The current study contributes to a growing understanding of how embodied learning unfolds during the early years. It affirms theories of sensitive periods and embodied cognition, suggesting that fine motor activities embedded in learning contexts can influence how neural and behavioral systems evolve [17,18]. When toddlers engage in shared reading, scribbling, and turning pages, they are not only building language and cognitive skills but also strengthening sensorimotor pathways critical for later coordination [29]. These routine actions, performed in a social and emotionally rich environment, may help establish integrated brain networks that support self-regulation, attention, and motor control [37]. By reinforcing the link between early experiences and motor function development, the present study supports the view that cognitive and motor domains are deeply interconnected from the start. Future studies should explore how the quality, frequency, and cultural context of early literacy practices influence these developmental pathways.

This study is not without its limitations. First, although our analysis draws from a rich, prospective longitudinal dataset, the measure of early literacy stimulation relied on parent-reported frequency of shared reading and related activities. Our self-reported literacy exposure index, while inspired from informal home literacy environment measures, does not have established construct validity and may be influenced by recall or social desirability biases. Nonetheless, parental perspectives offer important information about children’s early experiences and how these may contribute to long-term developmental outcomes. Second, while the longitudinal design enables us to trace associations between early experiences and later outcomes, the findings remain correlational, preventing any definitive causal conclusions. Third, although our motor outcomes were measured using standardized assessments by trained professionals, the scope of motor development captured in this study was limited to specific gross motor domains. It is possible that literacy stimulation more strongly predicts fine motor or graphomotor outcomes that were not assessed here. Future work might incorporate broader or more detailed motor assessments and explore how early literacy behaviors interact with emerging sensorimotor and cognitive pathways.

There are several chief strengths that enhance the relevance of this study. First, we use a prospective longitudinal design, which enables us to examine how early home literacy experiences predict developmental outcomes years later, offering valuable insight into early influences on motor development during a critical school transition. Second, data were drawn from a millennial birth cohort, all confounding variables were assessed prior to the predictor, which strengthens the temporal ordering of our analysis. Third, we accounted for the differing socialization experiences and developmental pathways of boys and girls, allowing for a more precise and inclusive understanding of how early literacy experiences relate to later motor outcomes. These features contribute to the study’s internal validity while offering meaningful insights that can inform intervention and educational practice. Together, these strengths enhance the study’s internal validity and practical relevance for developmental science and early childhood policy.

## 5. Conclusions

The present findings highlight the developmental value of literacy-related interactions in toddlerhood. Activities such as reading together, exploring writing tools, and engaging in storytelling offer rich opportunities for children to practice coordination and control in a meaningful context.

Importantly, sex-stratified results suggest that the developmental benefits of literacy experiences may differ for boys and girls. For girls, literacy stimulation was linked more strongly to motor outcomes, potentially reflecting early socialization patterns that encourage fine-motor and symbolic play. For boys, nonsignificant associations could stem from differences in early interests, parental influences, and existing motor activities typically preferred by boys. These distinctions underscore the need for future research to investigate how gender socialization and environmental context shape the interplay between early experiences and developmental outcomes.

To ensure equitable benefits, early childhood policies should expand access to books, culturally relevant materials, and parent–child programs, particularly for families from low socioeconomic backgrounds. Encouraging parents to integrate movement, touch, and gesture into literacy routines, such as acting out stories or tracing words, can support both cognitive and motor development. Early childhood education programs should also ensure that boys and girls alike are exposed to diverse literacy and play experiences that engage fine and gross motor skills. Health and education professionals can also contribute to encouraging routines that involve literacy practices, which include movement, touch, and gesture, fostering a full range of children’s developmental potential. When supported from the start, these small, everyday experiences can help shape lasting trajectories of engagement, confidence, and competence.

## Figures and Tables

**Table 1:** Descriptive statistics for study variables.

		Boys			Girls	
	M (SD)	Categorical Variables (%)	Range	M (SD)	Categorical Variables (%)	Range
Predictor (2 years)						
Literacy stimulation (2 years) 0 = No stimulation 1 = One type 2 = Two types 3 = Three types		1.8 8.6 34.9 54.7			0.2 4.5 37.9 57.3	
Outcomes (6 years)						
Motor control skills	8.71 (2.44)		2.00–15.00	9.05 (2.65)		1.00–17.00
Locomotion skills	8.97 (2.74)		1.00–17.00	9.63 (2.81)		1.00–17.00
Control variables						
Gestational smoking or substance use (5 months) 1 = Any smoking or substance use		31.8			31.1	
Premature birth (5 months) 1 = <37 weeks		5.2			4.2	
Weight for gestational age (5 months)	56.63 (8.62)		15.23–86.15	55.42 (7.89)		18.47–82.83
Mother antisocial behavior (5 months) 1 = Above the median		26.8			25.0	
Maternal education (5 months) 1 = Did not finish high school		15.4			15.2	
Family dysfunction (5 months) 1 = Above the median		47.0			46.2	
Maternal depressive symptoms (1.5 years) 1 = Above the median		45.4			43.7	
Child temperament problems (1.5 years) 1 = Above the median		48.5			48.5	
Family income (2 years) 1 = Insufficient		18.6			18.9	
Family configuration (2 years) 1 = Non-intact		14.9			13.0	
Child neurocognitive skills (2 years) 0 = Score of 0 1 = Score of 1 2 = Score of 2 3 = Score of 3		20.9 52.2 23.9 2.9			19.8 51.5 23.2 5.5	

Notes. M = mean; SD = standard deviation. Analyses corrected for attrition bias. Data were compiled from the final master file of the Quebec Longitudinal Study of Child Development (1998–2010), *©Gouvernement du Québec, Institut de la statistique du Québec*.

**Table 2:** Unstandardized regression coefficients (standard error) reflecting the adjusted relationship between baseline and concurrent child and family characteristics between ages 5 months and 2 years and literacy exposure at age 2 years [45].

	b (SE)	
	Literacy Exposure (2 years)	
	*Boys*	*Girls*
Gestational smoking or substance use (5 months)	0.03 (0.05)	−0.07 (0.04)
Premature birth (5 months)	0.16 (0.11)	−0.23 (0.12) *
Weight for gestational age (5 months)	0.00 (0.00)	0.00 (0.00)
Mother antisocial behavior (5 months)	0.00 (0.05)	0.05 (0.04)
Maternal education (5 months)	−0.18 (0.07) *	−0.08 (0.06)
Family dysfunction (5 months)	−0.09 (0.05)*	−0.07 (0.04)
Maternal depressive symptoms (1.5 years)	0.01 (0.05)	−0.10 (0.04) *
Child temperament problems (1.5 years)	−0.06 (0.05)	−0.04 (0.04)
Family income (2 years)	−0.01 (0.07)	−0.10 (0.06)
Family configuration (2 years)	−0.03 (0.08)	0.09 (0.06)
Child neurocognitive skills (2 years)	0.01 (0.03)	0.09 (0.02) ***
Adjusted R^2^	0.018 *	0.054 ***

Notes. * *p* ≤ 0.05, ** *p* ≤ 0.01, *** *p* ≤ 0.001. Analyses corrected for attrition bias. Data were compiled from the final master file of the Quebec Longitudinal Study of Child Development (1998–2010), *©Gouvernement du Québec, Institut de la statistique du Québec*.

**Table 3:** Unstandardized regression coefficients (standard error) reflecting the adjusted relationship between literacy exposure at age 2 years and motor development at age 6 years.

		Age 6 Years	
		Motor Control Skills	Locomotion Skills
**Boys**	*Literacy exposure (2 years)*	*0.22 (0.15)*	*0.29 (0.16)*
	Gestational smoking or substance use (5 months)	0.02 (0.26)	−0.11 (0.28)
	Premature birth (5 months)	0.30 (0.52)	−0.51 (0.49)
	Weight for gestational age (5 months)	0.02 (0.01)	0.02 (0.02)
	Mother antisocial behavior (5 months)	0.35 (0.24)	−0.02 (0.28)
	Maternal education (5 months)	−0.27 (0.37)	−0.62 (0.38)
	Family dysfunction (5 months)	−0.43 (0.24)	−0.53 (0.26) *
	Maternal depressive symptoms (1.5 years)	−0.09 (0.25)	0.00 (0.27)
	Child temperament problems (1.5 years)	−0.03 (0.22)	0.01 (0.25)
	Family income (2 years)	−0.91 (0.36) *	−0.93 (0.43) *
	Family configuration (2 years)	0.29 (0.36)	0.49 (0.46)
	Child neurocognitive skills (2 years)	−0.11 (0.15)	−0.01 (0.16)
	Adjusted R^2^	0.047 *	0.055 *
**Girls**	*Literacy exposure (2 years)*	0.46 (0.18) *	*0.36 (0.20)*
	Gestational smoking or substance use (5 months)	−0.08 (0.26)	−0.29 (0.28)
	Premature birth (5 months)	−0.30 (0.48)	0.60 (0.56)
	Weight for gestational age (5 months)	0.03 (0.02)	0.03 (0.02)
	Mother antisocial behavior (5 months)	−0.32 (0.28)	−0.03 (0.30)
	Maternal education (5 months)	−0.03 (0.35)	0.21 (0.39)
	Family dysfunction (5 months)	0.50 (0.24) *	0.04 (0.26)
	Maternal depressive symptoms (1.5 years)	0.05 (0.25)	0.00 (0.27)
	Child temperament problems (1.5 years)	0.43 (0.22)	−0.30 (0.24)
	Family income (2 years)	−0.87 (0.37) *	−0.70 (0.38)
	Family configuration (2 years)	0.09 (0.42)	0.12 (0.43)
	Child neurocognitive skills (2 years)	−0.02 (0.14)	0.22 (0.16)
	Adjusted R^2^	0.054 **	0.037 *

Notes. * *p* ≤ 0.05, ** *p* ≤ 0.01, *** *p* ≤ 0.001. Analyses corrected for attrition bias. Data were compiled from the final master file of the Quebec Longitudinal Study of Child Development (1998–2010), *©Gouvernement du Québec, Institut de la statistique du Québec*.

## Data Availability

The data presented in this study are available on request from the *Institut: de la Statistique du Québec (ISQ)*. The data are not publicly available due to permission of the ISQ.
